# Protective Effect of Statins on Pulmonary Hypertension in Chronic Obstructive Pulmonary Disease Patients: A Nationwide Retrospective, Matched Cohort Study

**DOI:** 10.1038/s41598-020-59828-0

**Published:** 2020-02-20

**Authors:** Wen-Ting Wu, Chung-Yu Chen

**Affiliations:** 1 Master Program in Clinical Pharmacy, School of Pharmacy, Kaohsiung Medical University, Kaohsiung, Taiwan; 2 Department of Pharmacy, Wan Fang Hospital, Taipei Medical University, Taipei, Taiwan; 3 Department of Pharmacy, Kaohsiung Medical University Hospital, Kaohsiung, Taiwan; 4 Department of Medical Research, Kaohsiung Medical University Hospital, Kaohsiung, Taiwan

**Keywords:** Peripheral vascular disease, Outcomes research

## Abstract

In Taiwan, patients with pulmonary hypertension (PH) related to chronic obstructive pulmonary disease (COPD) are most common PH population (group 3). However, efficacy of medical treatments and optimal prevention methods in this group remain uncertain. Statins such as indirect RhoA/Rho-kinase inhibitors influence one of key signalling pathways that promote PH onset. In this study, we explored protective effects of statins against PH in COPD patients using database from Taiwan National Health Insurance programme from 2002 to 2017. The main outcome was the risk of PH. The Cox proportional-hazards model and the Fine and Gray model were used to adjust covariate and competing risks to estimate the subdistribution hazard ratios (sHRs). 553,617 newly diagnosed COPD patients were stratified by statin users (n = 41,168) and statin nonusers (n = 512,449). After 1:1 propensity score matching of statin users (n = 41,163), and 41,163 statin nonusers were included for outcome analysis. Statin users had a 22% lower risk of PH than nonusers (sHR: 0.78, 95% confidence interval: 0.65–0.94). During subgroup analysis, taking higher daily doses and for a longer duration displayed a more significantly reduced risk of PH (both P for trend <0.001). Statins may have a protective effect against PH that is dose- and time-dependent.

## Introduction

Pulmonary hypertension (PH) is a pathophysiologic and hemodynamic condition that increases the pressure level in the pulmonary arteries, veins and/or capillaries. Eventually, under these conditions, the need for the heart ventricles to contract more and more to pump blood through the lungs and heart can result in heart failure, cardiovascular diseases, or respiratory function loss^1^. In 2018, the sixth World Symposium on PH (WSPH) released an updated statement separating PH patients into five groups according to clinical presentation, hemodynamic characteristics, pathophysiology, and therapeutic strategy^2^. The incidence of PH is about 2.4 cases per million adults per year in the United States^3–6^. One Taiwan prevalence survey suggested the patients with PH related to COPD (group 3 in the WSPH classification scheme) is the most common PH population in Taiwan^7^. Of interest, while the severity of PH among patients in group 3 may be less than that in other PH groups, the three-year survival rate in this group is the lowest across all five PH groups^8^. This lower survival rate might due to a lack of evidence and efficacy regarding the use of current medical treatments for PH including supportive therapy and certain drugs in PH related to COPD. Besides, there has been no clear prevention strategy revealed that can reduce the risk of PH in COPD patients; currently, only long-term oxygen therapy can improve symptoms. Given these facts, the development of a new treatment in this population is necessary^9,10^.

There are many studies that have explored the repurposing of PH treatment drugs on the market for other indications. For example, 3-hydroxy-3-methyl-glutaryl-coenzyme A reductase inhibitors are cholesterol-lowering drugs able to reduce the low-density lipoprotein cholesterol level by around 10% to 50% as well as the triglyceride concentration to a small degree. Because of their good lipid-lowering effects, statins are the first-choice therapeutic modalities in hyperlipidemia patients^11^. In addition to their lipid-lowering effects, statins also have displayed anti-inflammatory^12–14^, anti-proliferative^15–17^ and anti-thrombotic^18,19^ properties. These effects are associated with the pathophysiology of PH related to COPD. Moreover, statins are indirect RhoA/Rho-kinase inhibitors. Upon blocking the RhoA/Rho-kinase signalling pathway, these medications are capable of stopping vasoconstriction, endothelial nitric oxide synthase, cellular proliferation and apoptosis. However, to date, only *in vitro* studies have indicated that statins inhibit systemic inflammatory and pulmonary vascular proliferation, and block the RhoA/Rho-kinase signalling pathway; the efficacy of statins in human clinical trials remains unclear.

As such, we conducted a nationwide, population-based retrospective cohort study to explore whether the protective effects of statins could reduce the risk of PH in patients with COPD. Moreover, we compared the protective effects of different types of statins and examined whether such effects were dose- or time-dependent.

## Results

### Study population

**Figure 1:**
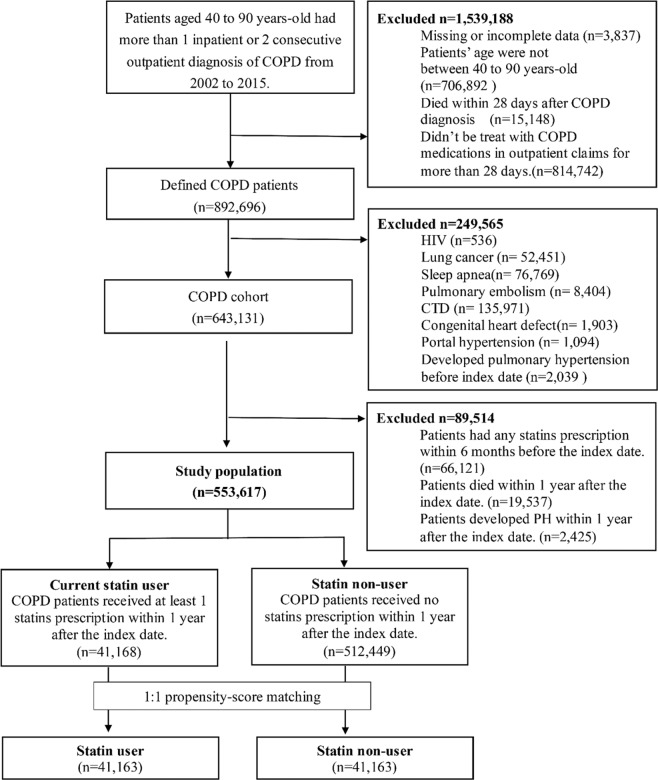
Result of flow chart in study population.

Based on the inclusion and exclusion criteria, a total of 553,617 patients were included in the newly diagnosed COPD cohort (Fig. 1). According to statin exposure, there were s 41,168 statin users and 512,449 nonusers of statins in study population. After 1:1 propensity score (PS) matching, we included 41,163 statin users and 41,163 statin nonusers for PH outcome analysis. Before PH matching, the mean age of patients in the user group (64.60 years) was slightly higher than that in the nonuser group (63.95 years). Because of the indications of statins, patients with statins displayed significantly higher rates of dyslipidemia (P < 0.001), coronary artery disease (P < 0.001), and ischaemic stroke (P < 0.001). Most of the comorbidities were found in significantly higher degrees in the user group than in the nonuser group, with the exception of interstitial pulmonary diseases, asthma and malignant and haemorrhagic stroke. In particular, rates of interstitial pulmonary diseases and malignant stroke were similar between the two groups (P = 0.543 and P = 0.250). Comedication use presented the same trend of comorbidity, while rates of severe and moderate exacerbations of COPD displayed a significant difference (P < 0.001) between the two user groups, although the majority of patients showed no exacerbation in their condition within one year after the index date. After PS matching, there was a significant difference noted in the distribution of comorbidities and concurrent medication use between the two groups. A Cox proportional-hazards (CPH) model was established to adjust all imbalanced characteristics in the following analysis. The details of baseline characteristics of the COPD cohort are presented in Table 1.

**Table 1:** Baseline characteristics of COPD patients before and after1-to-1propensity score matching, stratified according to statins using.

Characteristics n (%)	Before matching			After matching		
	User N = 41,168	Non-user N = 512,449	*p*-value	User N = 41,163	Non-user N = 41,163	*p*-value
Propensity score (SD)	0.88 (0.08)	0.93 (0.05)	<0.001	0.75 (0.03)	0.75(0.03)	1.000
**Age group**						
Mean (SD)	64.60 (11.06)	63.95 (12.85)	<0.001	64.60 (11.06)	64.67 (11.27)	0.370
40 ≤ age < 50	3,937 (9.56)	83,280 (16.25)	<0.001	3,936 (9.56)	3,936 (9.56)	1.000
50 ≤ age < 60	10,204 (24.79)	117,885 (23.00)		10,204 (24.79)	10,204 (24.79)	
60 ≤ age < 70	12,356 (30.01)	119,985 (23.41)		12,354 (30.01)	12,354 (30.01)	
70 ≤ age < 80	10,827 (26.30)	123,860 (24.17)		10,825 (26.30)	10,825 (26.30)	
80 ≤ age	3,844 (9.34)	67,439 (13.16)		3,844 (9.34)	3,844 (9.34)	
Male	23,303 (56.60)	313,271 (61.13)	<0.001	23,301 (56.61)	23,301 (56.61)	1.000
**Insurance premium (TWD)**						
≤22,800 TWD	21,581 (52.42)	280,896 (54.81)	<0.001	21,581 (52.43)	21,582 (52.43)	0.994
>22,800 TWD	19,587 (47.58)	231,553 (45.19)		19,582 (47.57)	19,581 (47.57)	
**Urbanization level**						
Urban	20,416 (49.59)	252,309 (49.24)	0.201	20,411 (49.59)	20,346 (49.43)	0.646
Suburban	16,255 (39.48)	202,886 (39.59)		16,255 (39.49)	16,237 (39.45)	
Rural	4,497 (10.92)	57,254 (11.17)		4,497 (10.92)	4,580 (10.13)	
**Comorbidity**						
Dyslipidemia	25,510 (61.97)	34,381 (6.71)	<0.001	25,510 (61.97)	4,603 (61.18)	<0.001
Hypertension	27,917 (67.81)	216,905 (42.33)	<0.001	27,912 (67.81)	27,916 (67.82)	0.976
Diabetes Mellitus	15,840 (38.48)	75,574 (14.75)	<0.001	15,835 (38.47)	16,043 (38.97)	0.137
Obesity	215 (0.52)	1,017 (0.20)	<0.001	215 (0.52)	142 (0.34)	<0.001
Chronic kidney disease	2,010 (4.88)	12,770 (2.49)	<0.001	2,010 (4.88)	1,668 (4.05)	<0.001
Chronic liver disease	4,892 (11.88)	49,970 (9.75)	<0.001	4,892 (11.88)	4,820 (11.71)	0.437
Arrhythmia	4,810 (11.68)	41,636 (8.12)	<0.001	4,805 (11.67)	4,739 (11.51)	0.472
Interstitial pulmonary diseases	177 (0.43)	2,310 (0.45)	0.543	177 (0.43)	161 (0.39)	0.383
Asthma	15,607 (37.91)	197,980 (38.63)	0.004	15,604 (37.91)	15,760 (37.29)	0.263
Malignant	4,236 (10.29)	53,653 (10.47)	0.250	4,235 (10.29)	4,369 (10.61)	0.127
**ASCVD**						
Coronary artery disease	13,622 (33.09)	76,755 (14.98)	<0.001	13,618 (33.08)	8,400 (33.41)	<0.001
Peripheral vascular disease	1,582 (3.84)	11,217 (2.19)	<0.001	1,582 (3.84)	1,260 (3.06)	<0.001
Ischemic stroke/TIA	6,239 (15.15)	45,500 (8.88)	<0.001	6,238 (15.15)	4,667 (15.34)	<0.001
Hemorrhagic stroke	583 (1.42)	8,447 (1.65)	<0.001	583 (1.42)	825 (1.20)	<0.001
Heart failure	6,155 (14.95)	42,883 (8.37)	<0.001	6,150 (14.94)	6,152 (14.95)	0.984
Left ventricular hypertrophy	435 (1.06)	3,154 (0.62)	<0.001	435 (1.06)	430 (1.04)	0.864
**Co-medication**						
Digoxin	1,902 (4.62)	17,049 (3.33)	<0.001	1,898 (4.61)	2,045 (4.97)	0.016
Oral anticoagulant agents	1,004 (2.44)	6,669 (1.30)	<0.001	1,003 (2.44)	790 (2.92)	<0.001
Oral antiplatelet agents	20,260 (49.21)	115,688 (22.58)	<0.001	20,257 (49.21)	13,406 (49.57)	<0.001
Diuretics	12,105 (29.40)	90,184 (17.60)	<0.001	12,100 (29.40)	10,509 (29.53)	<0.001
CCB	21,022 (51.06)	162,839 (31.78)	<0.001	21,019 (51.06)	19,511 (51.40)	<0.001
ACEI/ACB	21,653 (52.60)	134,311 (26.21)	<0.001	21,648 (52.59)	17,584 (52.72)	<0.001
Beta blocker	14,761 (35.86)	97,769 (19.08)	<0.001	14,757 (35.85)	11,917 (35.95)	<0.001
Metformin	11,085 (26.93)	44,553 (8.69)	<0.001	11,080 (26.92)	9,567 (26.24)	<0.001
Fibrate	4,875 (11.84)	14,045 (2.74)	<0.001	4,873 (11.84)	2,080 (11.05)	<0.001
Other lipid-lowering drugs	186 (0.45)	541 (0.11)	<0.001	186 (0.45)	64 (0.16)	<0.001
**Medication for COPD**						
LABA	933 (2.27)	11,143 (2.17)	0.220	933 (2.27)	859 (2.09)	0.077
LABA/ICS	4,126 (10.02)	48,234 (9.41)	<0.001	4,125 (10.02)	3,515 (10.54)	<0.001
LAMA	1,614 (3.92)	17,393 (3.39)	<0.001	1,614 (3.92)	1,187 (3.88)	<0.001
LABA/LAMA	214 (0.52)	1,814 (0.35)	<0.001	214 (0.52)	132 (0.32)	<0.001
SABA	3,261 (7.92)	42,497 (8.29)	0.008	3,260 (7.92)	3,244 (7.88)	0.836
SAMA	1,138 (2.76)	17,985 (3.51)	<0.001	1,137 (2.76)	1,447 (2.52)	<0.001
SABA/SAMA	1,588 (3.86)	19,001 (3.71)	0.123	1,588 (3.86)	1,407 (3.42)	0.001
Systemic beta-2 agonists	10,621 (25.80)	129,995 (25.37)	0.052	10,618 (25.80)	10,859 (25.38)	0.056
ICS	1,190 (2.89)	15,262 (2.98)	0.314	1,189 (2.89)	1,102 (2.68)	0.065
Methylxanthines	22,245 (54.03)	253,261 (49.42)	<0.001	22,241 (54.03)	21,248 (54.62)	<0.001
**COPD severity**						
**Moderate exacerbations**						
0	36,309 (88.20)	446,596 (87.15)	<0.001	30,446 (73.96)	30,522 (73.15)	0.799
1	3,385 (8.22)	41,982 (8.19)		4,480 (10.88)	4,469 (10.86)	
≥2	1,474 (3.58)	23,871 (4.66)		6,237 (15.15)	6,172 (15.99)	
**Severe exacerbations**						
0	30,446 (73.96)	371,050 (72.41)	<0.001	36,304 (88.20)	36,307 (88.20)	0.995
1	4,485 (10.89)	55,841 (10.90)		3,385 (8.22)	3,387 (8.23)	
≥2	6,237 (15.15)	85,558 (16.70)		1,474 (3.58)	1,469 (3.57)	

(COPD = chronic obstructive pulmonary disease; TWD = Taiwan dollars; ASCVD = atherosclerotic cardiovascular disease; TIA = Transient ischemic attack; CCB = calcium channel blocker; ACEI = angiotensin converting enzyme inhibitor; ARB = angiotensin receptor blocker; LABA = Long-acting β_2_-aginist; LAMA = Long-acting muscarinic antagonists; SABA = Short-acting β_2_-aginist; SAMA = Short-acting muscarinic antagonists; ICS = Inhaled corticosteroid).

#### Incidence

A total of 242 (0.59%) users and 338 (0.82%) nonusers of statins, respectively, experienced PH. The mean follow-up times in the user and nonuser groups were 4.13 and 4.28 years. The statin user group displayed a lower incidence rate of PH onset as compared with the nonuser group (1.43 vs. 1.97 per 1,000 person-years). The CPH model analysis findings are summarised in Table 2. In both a univariate analysis [crude hazard ratio (HR): 0.72, 95% confidence interval (CI): 0.61–0.85; P < 0.001) and multivariate analysis (adjusted HR (aHR): 0.76, 95% CI: 0.63–0.93; P = 0.006), a significantly lower HR of PH incidence between statin users and nonusers was observe. There were 6,997 (16.99%) statin users and 5,553 (13.49%) statin nonusers who died within the five-year study period. Death may be a competing risk for a PH event. To adjust the competing risk, we conducted a multivariate with competing risk analysis, where the incidence of PH still presented a significantly lower subdistribution HR (sHR) between statin users and nonusers (sHR: 0.78, 95% CI: 0.65–0.94; P = 0.010) The above results suggest that statins could reduce the risk of PH by approximately 28% to 22% and provide a protective effect against PH (Table 2).

**Table 2:** Multivariate cox proportional hazard model analysis for variables related to the risk of PH, stratified according to statins using (^a^Adjusted for age group, sex, income, comorbidity, co-medication, and COPD severity; ^b^Entry regression model after the stepwise multiple regression analysis; COPD = chronic obstructive pulmonary disease; PH = pulmonary hypertension; HR = hazard ratio; TWD = Taiwan dollars; ASCVD = atherosclerotic cardiovascular disease; TIA = Transient ischemic attack; CCB = calcium channel blocker; ACEI = angiotensin converting enzyme inhibitor; ARB = angiotensin receptor blocker; * < 0.05; ** < 0.01; *** < 0.001).

Variables	Crude	*p*-value	Adjusted	*p*-value	Subdistribution	*p*-value
	HR (95% CI)		HR^a^ (95% CI)		HR^a^ (95% CI)	
User *vs*. non-user	0.73 (0.61–0.86)^***^	<0.001	0.76 (0.63–0.93)^**^	0.007	0.78 (0.65–0.94)^*^	0.010
**Age group**^**b**^						
40 ≤ age < 50	1 (reference)		1 (reference)		1 (reference)	
50 ≤ age < 60	1.55 (0.95–2.53)	0.080	1.44 (0.88–2.35)	0.150	1.43 (0.87–2.34)	0.154
60 ≤ age < 70	2.79 (1.76–4.44)^***^	<0.001	2.05 (1.28–3.29)^**^	0.003	2.05 (1.27–3.30)^**^	0.003
70 ≤ age < 80	4.24 (2.68–6.70)^***^	<0.001	2.46 (1.53–3.94)^***^	<0.001	2.35 (1.44–3.82)^**^	0.001
80 ≤ age	6.53 (4.04–10.55)^***^	<0.001	2.98 (1.80–4.91)^***^	<0.001	2.45 (1.46–4.13)^**^	0.001
Male^b^	1.19 (1.01–1.41)^*^	0.037	1.24 (1.05–1.47)^*^	0.012	1.22 (1.03–1.45)^*^	0.020
**Insurance premium (NT$)**^**b**^						
≤22,800	1 (reference)		1 (reference)		1 (reference)	
>22,800	0.48 (0.41–0.58)^***^	<0.001	0.74 (0.61–0.89)^**^	0.002	0.80 (0.66–0.96)^*^	0.019
**Urbanization level**						
Urban	1 (reference)		1 (reference)		1 (reference)	
Suburban	1.37 (1.15–1.63)^***^	<0.001	1.23 (1.03–1.46)^*^	0.022	1.25 (1.05–1.49)^*^	0.011
Rural	1.18 (0.89–1.55)	0.250	0.96 (0.72–1.26)	0.753	0.97 (0.74–1.29)	0.851
**Comorbidity**						
Dyslipidemia	0.59 (0.49–0.71)^**^	<0.001	0.84 (0.67–1.04)	0.113	0.86 (0.69–1.07)	0.178
Hypertension	1.32 (1.10–1.58)^*^	0.003	0.87 (0.70–1.08)	0.214	0.87 (0.69–1.09)	0.225
Diabetes Mellitus	1.05 (0.89–1.24)	0.575	1.11 (0.90–1.38)	0.320	1.05 (0.85–1.30)	0.666
Obesity	1.54 (0.58–4.13)	0.387	2.23 (0.83–5.99)	0.112	2.30 (0.85–6.21)	0.100
Chronic kidney disease	2.06 (1.50–2.84)^**^	<0.001	1.19 (0.86–1.65)	0.292	1.03 (0.74–1.44)	0.857
Chronic liver disease	0.85 (0.65–1.12)	0.244	0.91 (0.69–1.19)	0.496	0.89 (0.68–1.17)	0.409
Arrhythmia^b^	2.93 (2.43–3.52)^***^	<0.001	1.50 (1.22–1.85)^***^	<0.001	1.49 (1.20–1.84)^***^	<0.001
Interstitial pulmonary diseases	3.39 (1.51–7.57)^**^	0.003	2.15 (0.96–4.84)	0.063	1.99 (0.88–4.47)	0.098
Asthma^b^	2.00 (1.68–2.39)^***^	<0.001	1.65 (1.38–1.98)^***^	<0.001	1.72 (1.43–2.06 ^***^	<0.001
Malignant	0.84 (0.62–1.13)	0.237	0.83 (0.61–1.11)	0.210	0.78 (0.57–1.05)	0.098
**ASCVD**						
Coronary artery disease	1.62 (1.37–1.92)^***^	<0.001	0.87 (0.72–1.06)	0.179	0.87 (0.71–1.06)	0.165
Peripheral vascular disease	1.02 (0.65–1.61)	0.933	0.74 (0.47–1.17)	0.193	0.70 (0.44–1.12)	0.137
Ischemic stroke/TIA	1.10 (0.87–1.40)	0.432	0.75 (0.59–0.97)^*^	0.026	0.70 (0.55–0.90)^**^	0.006
Hemorrhagic stroke	0.72 (0.32–1.60)	0.414	0.53 (0.24–1.19)	0.123	0.49 (0.22–1.09)	0.080
Heart failure^b^	5.32 (4.45–6.37)^***^	<0.001	2.17 (1.74–2.71)^***^	<0.001	2.12 (1.67–2.67)^***^	<0.001
Left ventricular hypertrophy	2.63 (1.58–4.39)^***^	<0.001	1.21 (0.72–2.04)	0.464	1.23 (0.73–2.09)	0.443
**Co-medication**						
Digoxin^b^	5.06 (4.10–6.25)^***^	<0.001	1.50 (1.17–1.93)^**^	0.001	1.43 (1.11–1.85)^**^	0.006
Oral anticoagulant agents	2.96 (2.05–4.27)^***^	<0.001	1.01 (0.69–1.50)	0.947	1.01 (0.68–1.49)	0.974
Oral antiplatelet agents	1.69 (1.44–1.99)^***^	<0.001	1.12 (0.92–1.36)	0.275	1.12 (0.91–1.36)	0.285
Diuretics^b^	3.83 (3.19–4.61)^***^	<0.001	1.97 (1.59–2.44)^***^	<0.001	1.94 (1.57–2.39)^***^	<0.001
CCB	1.22 (1.04–1.44)^***^	<0.001	0.93 (0.77–1.11)	0.412	0.93 (0.77–1.13)	0.465
ACEI/ACB	1.75 (1.48–2.07)^***^	<0.001	1.20 (0.99–1.46)	0.065	1.22 (1.00–1.48)	0.050
Beta blocker	1.14 (0.96–1.35)	0.134	0.92 (0.76–1.10)	0.337	0.92 (0.77–1.11)	0.389
Metformin	0.74 (0.60–0.91)^**^	0.004	0.72 (0.55–0.93)^*^	0.011	0.73 (0.57–0.94)^*^	0.015
Fibrate	0.56 (0.39–0.81)^**^	0.002	0.81 (0.55–1.18)	0.275	0.82 (0.56–1.20)	0.313
**COPD severity**						
**Moderate exacerbations**^**b**^						
0	1 (reference)		1 (reference)		1 (reference)	
1	2.71 (2.15–3.41)^***^	<0.001	1.57 (1.23–2.01)^***^	<0.001	1.55 (1.19–2.02)^**^	0.001
≥2	2.77 (2.25–3.42)^***^	<0.001	1.49 (1.17–1.89)^**^	0.001	1.42 (1.11–1.83)^**^	0.006
**Severe exacerbations**^**b**^						
0	1 (reference)		1 (reference)		1 (reference)	
1	3.14 (2.53–3.89)^***^	<0.001	1.57 (1.23–1.99)^***^	<0.001	1.45 (1.13–1.87)^**^	0.004
≥2	4.84 (3.64–6.43)^***^	<0.001	1.71 (1.23–2.37)^**^	0.001	1.33 (0.96–1.85)	0.089

Statin users of an older age; male gender; with lower insurance premiums; who were living in the suburbs; and/or who had a cardiac arrhythmia, asthma or heart failure presented a significantly higher risk of developing PH as compared with nonusers. In contrast, patients with ischaemic stroke or transient ischaemic attack had a lower risk of PH (aHR; 0.75, 95% CI: 0.59–0.97; P = 0.026) Regarding the COPD severity in sHR analysis, only moderate exacerbation and one-time severe exacerbation significantly increased the risk of PH. We next conducted a stepwise multiple regression analysis to elucidate the main factors affecting the incidence rate of PH. Here, age group, gender, insurance premium, arrhythmia, asthma, heart failure, digoxin, diuretic and COPD severity displayed a significantly different risk. These significant factors were subsequently used to construct an adjusted model for use in the following subgroup analysis (Table 2).

#### Subgroup analysis

The most used statin was atorvastatin, with nearly 40% of statin users taking this medication. As indicated in Table 3, most of the statin used showed a trend of a low risk of PH except lovastatin (aHR: 1.63, 95% CI: 0.95–2.79; P = 0.076) Among all of those being used, pravastatin had the lowest aHR and appeared to significantly reduce the risk of PH by 56% (aHR: 0.44, 95% CI: 0.23–0.86; P = 0.016). After adjusting for all-cause mortality as a competing risk, the trend of the protective effect of each statin did not change.

**Table 3:** Subgroup analysis of risk of PH in different kind of statins, stratified according to statins using (^a^Adjusted for age group, gender, insurance premium, arrhythmia, asthma, heart failure, digoxin, diuretics, and COPD severity; HR = hazard ratio; PH = pulmonary hypertension; PY = person-year; Rate = (event/person-year) *1000; * < 0.05; ** < 0.01; *** < 0.001).

Kind of statins	User n = 41163				Non-user n = 41163			Crude	*p*-value	Adjusted	*p*-value	Subdistribution	*p*-value
	N	Events	Total of PY	Rate	Events	Total of PY	Rate	HR (95% CI)		HR^a^ (95% CI)		HR^a^ (95% CI)	
simvastatin	6575	42	28897.13	1.45	56	27522.95	2.03	0.72 (0.48–1.07)	0.102	0.66 (0.44–1.00)	0.050	0.71 (0.47–1.06)	0.094
lovastatin	4385	37	19622.88	1.89	22	18513.47	1.19	1.60 (0.94–2.71)	0.082	1.63 (0.95–2.79)	0.076	1.64 (0.97–2.77)	0.063
pravastatin	2588	13	10740.20	1.21	28	10820.43	2.59	0.47 (0.24–0.90)^*^	0.023	0.44 (0.23–0.86)^*^	0.016	0.45 (0.23–0.89)^*^	0.021
fluvastatin or pitavastatin	4084	19	15870.42	1.20	27	17050.70	1.58	0.76 (0.42–1.36)	0.351	0.74 (0.41–1.35)	0.331	0.78 (0.43–1.39)	0.391
atorvastatin	16331	92	66581.49	1.38	148	67789.98	2.18	0.63 (0.49–0.82)^**^	0.001	0.63 (0.23–0.82)^**^	0.001	0.65 (0.50–0.89)^**^	0.001
rosuvastatin	7200	39	28087.20	1.39	57	30232.80	1.89	0.74 (0.49–1.11)	0.142	0.75 (0.49–1.13)	0.167	0.77 (0.51–1.17)	0.227

A classification scheme for cumulative defined daily dose (cDDD) including seven levels was included in a multivariate CPH model analysis. The statin nonuser group was employed as a reference group in this analysis. Table 4 revealed that patients with higher cDDDs had lower aHR values of from 1.36 to 0.26 (P for trend ≤0.001). Further, among those patients using more than 180 cDDD, a significantly lower risk of PH was observed (aHR: 0.58, 95% CI: 0.42–0.81; sHR: 0.66, 95% CI: 0.47–0.92). The duration of statin use was calculated by year and divided into six categories. Patients with longer durations of statin use had a lower risk of PH (aHR: 1.15–0.31; P for trend ≤0.001), with a significantly lower risk of PH observed among those using statins for more than one year (aHR: 0.44, 95% CI: 0.31–0.64; sHR: 0.47, 95% CI: 0.32–0.67). Separately, we divided the frequency of use of statins into four levels and conducted a multivariate CPH model analysis. The most common frequency of statin use is 30 defined daily doses (DDDs) per month. Patients using greater numbers of DDDs of statins per month had a lower risk of PH (aHR: 0.81–0.51; P for trend ≤0.001). Further, patients who used more than 20 DDDs of statin per month started to show a significantly lower risk of PH (aHR: 0.51, 95% CI: 0.39–0.67; sHR: 0.54, 95% CI: 0.41–0.71).

**Table 4:** Subgroup analysis of risk of PH, stratified according to classification of cDDD, duration of statins use and intensity (^a^Adjusted for age group, gender, insurance premium, arrhythmia, asthma, heart failure, digoxin, diuretics, and COPD severity.; HR = hazard ratio; COPD = chronic obstructive pulmonary disease; PH = pulmonary hypertension; cDDD = cumulative defined daily doses; PY = person-year; Rate = (event/person-year) *1000; * < 0.05; ** < 0.01; *** < 0.001).

Group	N	Events	Total of PY	Rate	Crude	*p*-value	Adjusted	*p*-value	Subdistribution	*p*-value
					HR (95% CI)		HR^a^ (95% CI)		HR^a^ (95% CI)	
**cDDD**										
Non-user	41163	338	171937.85	1.97	1 (reference)		1 (reference)		1 (reference)	
cDDD < 28	4992	61	19423.87	3.14	1.60 (1.21–2.09)^**^	0.001	1.36 (1.03–1.79)^*^	0.031	1.36 (1.02–1.82)^*^	0.038
28 ≤ cDDD < 90	7147	59	26901.31	2.19	1.11(0.84–1.47)	0.450	1.06 (0.80–1.40)	0.672	1.11 (0.83–1.48)	0.489
90 ≤ cDDD < 180	6221	43	23988.18	1.79	0.91 (0.66–1.25)	0.560	0.89 (0.65–1.23)	0.479	0.94 (0.67–1.30)	0.695
180 ≤ cDDD < 365	8472	40	34531.87	1.16	0.59 (0.42–0.82)^**^	0.002	0.58 (0.42–0.81)^**^	0.001	0.66 (0.47–0.92)^*^	0.015
365 ≤ cDDD < 730	8495	25	37496.93	0.67	0.34 (0.23–0.51)^***^	<0.001	0.37 (0.24–0.55)^***^	<0.001	0.43 (0.28–0.65)^***^	<0.001
730 ≤ cDDD	5836	14	27464.22	0.51	0.26 (0.15–0.44)^***^	<0.001	0.26 (0.15–0.45)^***^	<0.001	0.32 (0.19–0.55)^***^	<0.001
*p* for trend test	<0.001									
**Duration of statins use (year)**										
Non-user	41163	338	171929.9123	1.97	1 (reference)		1 (reference)		1 (reference)	
year < 0.5	13448	125	51095.84931	2.45	1.24 (1.01–1.52)^*^	0.039	1.15 (0.93–1.41)	0.203	1.12 (0.91–1.38)	0.301
0.5 ≤ year < 1	7249	45	27638.78082	1.63	0.83 (0.61–1.13)	0.229	0.81 (0.59–1.10)	0.178	0.80 (0.58–1.09)	0.157
1 ≤ year < 2	8931	32	37213.73973	0.86	0.44 (0.30–0.63)^***^	<0.001	0.44 (0.31–0.64)^***^	<0.001	0.47 (0.32–0.67)^***^	<0.001
2 ≤ year < 3	5125	22	23182.96986	0.95	0.48 (0.31–0.74)^**^	0.001	0.51 (0.33–0.79)^**^	0.002	0.57 (0.37–0.88)^*^	0.011
3 ≤ year	6410	18	30677.67397	0.59	0.30 (0.19–0.48)^***^	<0.001	0.31 (0.19–0.49)^***^	<0.001	0.35 (0.22–0.56)^***^	<0.001
*p* for trend test	<0.001									
**Intensity (cDDD/month)**										
Non-user	41163	338	171929.9123	1.97	1 (reference)		1 (reference)		1 (reference)	
<10	4021	32	15812.85206	2.02	1.03 (0.72–1.48)	0.884	0.81 (0.56–1.16)	0.252	0.79 (0.55–1.15)	0.216
10 ≤ intensity < 20	21619	149	91493.42192	1.63	0.83 (0.68–1.01)	0.056	0.83 (0.69–1.01)	0.064	0.87 (0.71–1.05)	0.142
20≤	15523	61	62502.73973	0.98	0.50 (0.38–0.65)^***^	<0.001	0.51 (0.39–0.67)^***^	<0.001	0.54 (0.41–0.71)^***^	<0.001
*p* for trend test	<0.001									

#### Sensitivity analysis

As compared with the original definition of a PH event, a more precise definition did not change the trend of observing a protective effect against PH. Moreover, the sensitivity analysis had a lower risk of PH when compared with the original definition of a PH event in the statin user group (aHR: 0.70, 95% CI: 0.56–0.87 vs. aHR: 0.76, 95% CI: 0.63–0.93) Besides, extending the one-year confirmation period to three years and conducting a longer or shorter duration of observation did not have much of an influence on the outcome of PH risk. Findings of the sensitivity analysis are shown in Supplementary Tables 1 and 2.

## Discussion

During the five-year study observation period, the statin user group displayed a lower incidence rate of PH as compared with in the nonuser group (1.43 vs. 1.97 per 1,000 person-years; P < 0.001). After adjusting for age, sex, income, comorbidity, comedication use, COPD severity and competing risks, the finding was that statin use reduced the incidence rate of PH by 22% among COPD patients (sHR: 0.78, 95% CI: 0.65–0.94; P = 0.010). These results support the suggestion that statins offer a protective effect against PH in COPD patients.

The pathophysiology in PH related to COPD is complex and caused by multiple mechanisms. Pulmonary vascular remodelling, parenchymal lung destruction and hypoxia are the three known main independent mechanisms in PH related to COPD. Among these, pulmonary vascular remodelling and parenchymal lung destruction are observed early on during the course of PH. These conditions may also be associated with findings of systemic inflammation and endothelial cell dysfunction that are involved in the pathophysiology of COPD as well. If COPD continues to deteriorate toward hypoxemia, the onset of hypoxia causes pulmonary arterial vasoconstriction^9^. The protective effect of statins may be attributed to their anti-inflammatory effect that functions by limiting immune cell activation and reducing inflammatory cytokines^12–14^. A controlled pilot study showed that atorvastatin treatment in COPD patients significantly reduced the neutrophil count in sputum by 34% and the CD45+ cell count by 57% in lung biopsies (P = 0.008)^13^. Moreover, statins are the same as endothelin-1 receptor antagonists, which are specific drugs used for PH treatment in that endothelin-1 receptor antagonists are indirect RhoA/Rho-kinase inhibitors^20^. RhoA is one of the Rho G proteins, which are intracellular messengers. In the RhoA/Rho-kinase signalling pathway, RhoA activates the downstream effectors Rho-kinase I (ROCK-I) and Rho-kinase II (ROCK-II) and causes vasoconstriction, endothelial nitric oxide synthase, cellular proliferation and apoptosis. The expression of the signalling pathway is common in hypoxic lungs. Statins can decrease the progression of an early process in the cholesterol biosynthetic pathway as well as inhibit the synthesis of isoprenoids, which are prerequisite posttranslational lipid attachments necessary for Rho activation^20^. Statins also have anti-thrombotic effects: they inhibit the platelet-derived growth factor signal and reduce platelet thrombus formation in patients with idiopathic pulmonary arterial hypertension. Fluvastatin has shown beneficial effects on chronic hypoxia-induced PH by limiting endothelial nitric oxide synthase activity^18,19^. In summary, because of their role as indirect RhoA/Rho-kinase inhibitors and other pharmacological activities, we hypothesised that statins may have potential therapeutic benefits in PH.

To our knowledge, this study was the first to investigate whether statins can reduce the incidence of PH in the COPD population. The only other similar study involving patients with severe COPD supported that statin use is associated with a significantly lower PAWP (12 ± 5 vs. 15 ± 6 mmHg; P = 0.002) and PAPm (26 ± 7 vs. 29 ± 7 mmHg; P = 0.002) outcomes^21^. Findings of PAWP values over 15 mmHg and PAPm values over 20 mmHg lead to placement in the WSPH’s group 3. So, the reduction of PAWP and PAPm via statin use may support that statins have an association with the reduction of PH incidence. In this manner, these results complement our study findings. However, the incidence of group 3 PH is still unknown. One study reported an incidence of 14% among elderly patients older than 65 years, while the ASPIRE registry data showed that 56.7% of COPD patients had group 3 PH^8,22^. It is difficult to compare the incidence and prevalence rates among these studies and our research because the study populations vary. In our multiple regression analysis, older age, male gender, lower insurance premiums, living in the suburbs, having an arrhythmia, having asthma, having heart failure and COPD severity were4 risk factors for PH development. According to the pathophysiology and disease progression of PH, these risk factors appear reasonable^9^. However, patients with ischaemic stroke or transient ischaemic attack had a lower risk of PH (aHR: 0.75, 95% CI: 0.59–0.97; P = 0.026) The reasoning for this perhaps is that patients who experience ischaemic stroke have a ninefold higher mortality rate as compared with patients without stroke: in other words, they die before PH occurs, leading to a lower reported incidence of PH^23^. After adjusting the all-cause mortality as a competing risk, statin users still showed a significantly lower risk of PH in comparison with nonusers (sHR: 0.78, 95% CI: 0.65–0.94; P = 0.010).

During subgroup analysis, the results indicated dose- and time-dependent effects existed for statin use in that the statins achieved the protective effect against PH until patients achieved greater than 180 cDDD, reached one year of use, or used more than 20 DDDs per month. Among patients with greater cDDDs and longer durations of use, a lower risk of PH incidence could be observed. To understand the association between cDDD and the duration of statin use, this study completed a subgroup analysis of intensity, referring to the cDDD per month. The normal cDDD per month is 30 DDDs. However, the DDD is an average maintenance dose per day, so it may represent a moderate statin dose according to the drug’s lipid-lowering effects^11,24^. Patients who used more than 20 DDDs of statin per month displayed a significantly lower risk of PH (sHR: 0.54, 95% CI: 0.41–0.71). The exploration of the dose- and time-dependent effects of statins has been common in studies involving different populations, making the results more robust^25–27^.

The study is the first to explore the protective effects of statins against PH in COPD patients. In the study design, many important strengths can be observed. The Nation Health Insurance (NHI) database is one of the largest and most comprehensive medical population databases in the world, so the study had access to a larger sample size than other studies. Specifically, 41,163 COPD patients and 1,325 patients with PH related to COPD were included in the final analysis of this study. This is in comparison with other investigations, where the sample size of patients with PH related to COPD was only 40 to 60 patients^28–33^. This extensive sample size may provide enough power for statistical analysis. Another strength is the long observation time. The study relied on the database from 2002 to 2017 and included a five-year observation period, ensuring the availability of enough PH events for analysis. Further, the results were robust across several different definitions of statin drug exposure and observation duration.

In contrast, however, this study also had several limitations. The study population and outcomes were all defined based on the International Classification of Diseases, ninth revision, clinical modification (ICD-9-CM) or the International Classification of Diseases, 10th revision, clinical modification in the admissions record rather than on clinical diagnosis because the NHI database lacked examination results and laboratory data. Therefore, the definition of PH in this study was established according to a Chang *et al*. study that used the same NIH database. This previous study verified the accuracy of the diagnosis code (i.e., ICD-9-CM) by chart review in a medical centre hospital. The positive predictive value of using diagnosis codes in this regard was reported as up to 94.9%^7^. Then, the current study also used the second definition that combined the diagnosis codes and examinations to enhance the correct rate of PH diagnosis. Such examinations like right-heart catheterisation, echocardiographic, and chest X-ray were recommended by the 2015 European Society of Cardiology/European Respiratory Society guidelines for the diagnosis and treatment of pulmonary hypertension^10^. The limitations of the NHI database also include no access to smoking status, lifestyle, lung function or PH severity. The study also could not confirm that patients were taking treatments for PH or not. The reason for this is that patients in this population often do not fit the payment rules of PH-specific therapy in the NHI programme. So, many used those drugs at their own expense, which would not be recorded in the NIH database.

In conclusion, statins may have a protective effect against PH through reducing the incidence of PH in patients with COPD. Moreover, the protective effect was dose- and time-dependent. An age of more than 60 years, male gender, low income, heart failure, arrhythmia, asthma and COPD severity were risk factors for PH. However, we could not identify a consistent benefit in protective effect between different kinds of statins. Further randomised controlled trials involving different statins and accurate statin exposure control are required.

## Methods

### Data sources

Taiwan established the NHI programme in 1995. The Taiwan NIH programme, which covers 99.6% of 23 million Taiwanese and 93% of hospitals, clinics and pharmacies, represents one of the largest and most comprehensive medical population databases in the world. We used the full population database with all its medical records and multiple cause of death datasets from the NHI programme from 2002 to 2017^34^. To maintain the privacy of patients, the identity numbers of patients, medical institutions and medical providers were encrypted through the application of anonymous codes. All researchers in Taiwan are required to follow the Computer-processed Personal Data Protection Law and should not attempt to decrypt and impair the privacy of patients. We independently conducted this study at a subcenter of the Health and Welfare Data Science Centers at Kaohsiung Medical University. This study was approved by the institutional review board of Kaohsiung Medical University Chung-Ho Memorial Hospital (KMUHIRB-EXEMPT(I)-20190032).

### Study population

We identified newly diagnosed COPD patients from both outpatient and inpatient visit records from January 1, 2002 to December 31, 2015 (ICD-9-CM codes 490, 491, 492 and 496). Patients with more than one inpatient diagnosis or more than two consecutive outpatient diagnoses of COPD and who were treated using COPD medications according to outpatient claims for more than 28 days within one year after the primary COPD diagnosis date were defined as COPD patients. Patients with other etiologies of PH^10^ (Supplementary Table 3) and lung cancer (ICD-9-CM code 162) were excluded (n = 249,565). Patients who developed PH before their COPD diagnosis (n = 2,425) or died within 28 days after their COPD diagnosis (n = 15,148) were also excluded. The date of the primary diagnosis of COPD was defined as the index date.

### Baseline characteristics and COPD severity

Baseline characteristics and COPD severity were confirmed by medical records in the one year after the index date according to ICD-9-CM codes or ICD-10-CM codes. The demographic data contained age, age group, gender, urbanisation level^35,36^ and insurance premium information. To adjust for other confounders that might influence the outcome, we listed comorbidities including a high risk of atherosclerotic cardiovascular disease (ASCVD)^24^ and other medications used in the treatment of PAH [e.g., digoxin, calcium channel blockers, warfarin]. Detailed baseline characteristics and definitions are shown in Supplementary Table 4. The assessment of exacerbation risk in the Global Initiative for Chronic Obstructive Lung Disease (GOLD) guidelines was used to define the severity of COPD. The category of severe exacerbations refers to exacerbations leading to emergency room or hospital admission, while moderate exacerbations were those not leading to hospital admission but where patients were treated with SABA plus antibiotics or oral corticosteroids^37^. Because the index date was the first COPD diagnosis date, the severity of COPD was confirmed at one year after the index date.

The baseline characteristics of the original cohort were used to perform PS matching by multivariate logistic regression (via OneToManyMTCH, a SAS procedure; SAS Institute, Cary, NC, USA). Data on age group, gender, insurance premium, urbanisation, hypertension, diabetes, heart failure, left ventricular hypertrophy and COPD severity were used to calculate the propensity score. We adopted a 1:1 matching scheme to generate the statin user and nonuser groups for analysis after follow-up^38^.

#### Exposure and outcomes assessment

For the statin exposure definition, to solve the issue of reverse causality, we established a six-month drug washout period according to the pharmacokinetic and pharmacodynamic properties of statins^26,27^. Patients with any level of statin exposure during the washout period were excluded. Patients needed to use at least one statin in the one year after COPD diagnosis to be defined as statin users. Conversely, patients who never received prescriptions for statins in the one year after COPD diagnosis were defined as statin nonusers.

The incidence of PH was the primary outcome in the study. To identify the event of PH, we used the diagnosis code (i.e., 416.0, 416.8, or 416.9 in ICD-9-CM; I270, I272, I278, or I279 in ICD-10-CM) in the medical records to define the event of PH. Then, we applied two criteria to confirm patients truly had PH. For the first one, patients were required to have more than one inpatient diagnosis or emergency room admission for PH or more than two consecutive outpatient diagnoses of PH in one year. For the second, in the sensitivity analysis, patients had to undergo diagnostic examinations for PH (e.g., right-heart catheterisation, echocardiography, or chest X-ray) and receive a diagnosis of PH at the same time. We used the first criterion in the main analysis^4,7^.

To assess the influence of incidence among different kinds of statins, doses and time frames, this study included subgroup analyses. There were seven statins that were prescribed in Taiwan during the study period. The patients’ statin group was determined based on their most-used statin during the five-year observation period. We analysed the dose-dependent effect by considering the DDD, which was established by the World Health Organization to standardise the doses of different kinds of statins. The cDDD was the total amount of statin exposure during the five-year observation period and was divided into six levels (i.e., ≤28, 28–90, 90–180, 180–365, 365–730, and ≥730 cDDD). Additionally, the duration of statin use was calculated by year and divided into five levels (i.e., ≤0.5, 0.5–1, 1–2, 2–3, and ≥3 years). The intensity was calculated by dividing the cDDD by the duration of statin use during the whole five-year observation period. Then, we divided such into three levels to compare weather the intensity could affect the protective effect of statins^39^ (i.e., ≤10, 10–20, and ≥20 cDDD/month).

#### Follow-up time

**Figure 2:**
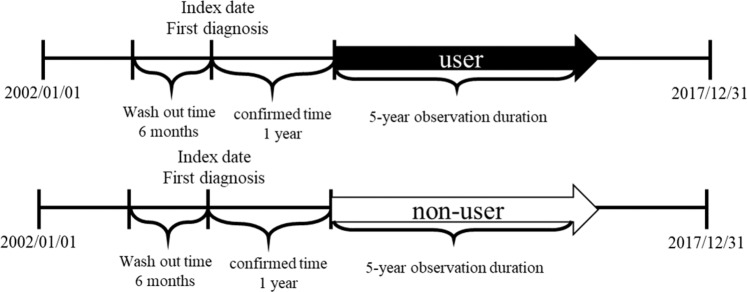
We set a five-year observation duration to reduce the influence of switching to different statins or discounting taking statins. The follow-up start form one year after index date and to the five years after index date.

The five-year follow-up period began at one year after the index date. Users and nonusers alike stopped participating at the occurrence of PH or censoring. If patients died before the occurrence of PH or were not diagnosed with PH by the end of the observation period, they were defined as being censors. The definition of censors was the same in both the user and nonuser groups. During the observation period, any statin exposure would not change the user group (Fig. 2).

#### Statistical analysis

For baseline characteristics, continuous variables were presented as means (standard deviations) and categorical variables were presented as percentages. Continuous variables were analysed by Student’s t-test or analysis of variance and categorical variables were analysed by Fisher’s exact test or chi-squared test if the value was less than 30. The crude incidence of PH was estimated as the total number of events during the five-year observation period divided by total person-years. This study conducted a CPH model to estimate the HR between the two user groups. The multivariate model was adjusted according to demographic characteristics, gender, comorbidities, comedication use and COPD severity to calculate the aHR. To reduce the influence of interaction and collinear effects between characteristics, stepwise selection was performed to select important factors to construct the multivariate regression model for subgroup analysis. Because of the competing risk of death, the Fine and Gray competing risk model was used to estimate the sHR in the primary outcome^40^.

All of the above analyses were performed using the SAS version 9.4. software program (SAS Institute, Cary, NC, USA). Statistical significance was determined as two-tailed and α = 0.05.

## Supplementary information

Supplementary information
