# Supplementary material for: Protective Effect of Statins on Pulmonary Hypertension in Chronic Obstructive Pulmonary Disease Patients: A Nationwide Retrospective, Matched Cohort Study
Source: Sci Rep. 2020 Feb 20;10:3104. doi: 10.1038/s41598-020-59828-0 (PMC7033169; doi:10.1038/s41598-020-59828-0)
Supplement: Supplementary file 1 — Supplementary information [file 41598_2020_59828_MOESM1_ESM.docx]

Protective Effect of Statins on Pulmonary Hypertension in Chronic Obstructive Pulmonary Disease Patients: A Nationwide Retrospective, Matched Cohort Study

**Wen-Ting Wu**1**, Chung-Yu Chen***1**,**

1 Department of Pharmacy, Kaohsiung Medical University Hospital, No. 100, Tzyou 1st Road, Sanmin District, Kaohsiung 80708, Taiwan

*corresponding: Prof. Chung-Yu, Email: [jk2975525@cc.kmu.edu.tw](mailto:jk2975525@cc.kmu.edu.tw)

Supplementary Table 1 Sensitivity analysis of risk of PH in definition of PH event, stratified according to statins using

| **Definition** | **User**  **n=41163** | | | **Non-user**  **n=41163** | | | **Crude** | ***p*-value** | **Adjusted** | ***p*-value** | **Subdistribution** | ***p*-value** |
| --- | --- | --- | --- | --- | --- | --- | --- | --- | --- | --- | --- | --- |
|  | **Event** | **Total of PY** | **Rate** | **Event** | **Mean of PY** | **Rate** | **HR (95% CI)** |  | **HR^a^ (95% CI)** |  | **HR^a^ (95% CI)** |  |
| Original | 242 | 170003.19 | 1.43 | 338 | 172061.34 | 1.97 | 0.72 (0.61-0.85)^***^ | <.001 | 0.76 (0.63-0.93)^**^ | 0.006 | 0.78 (0.65-0.94)^*^ | 0.010 |
| PH with exams | 179 | 169809.01 | 1.05 | 276 | 171929.91 | 1.61 | 0.66 (0.54-0.79)^***^ | <.001 | 0.70 (0.56-0.87)^**^ | 0.002 | 0.68 (0.56-0.82)^***^ | <.001 |

^a^ Adjusted for age group, sex, income, comorbidity, co-medication, and COPD severity.; *<0.05; **<0.01; ***<.001;

PH=pulmonary hypertension; HR=hazard ratio; PY=person-year; Rate= (event/person-year) *1000

Supplementary Table 2. Sensitivity analysis of risk of PH in one-year and three-year confirmed periods during different follow-up period, stratified according to statins using

^a^ Adjusted for age group, sex, income, comorbidity, co-medication, and COPD severity.; *<0.05; **<0.01; ***<.001;

PH=pulmonary hypertension; HR=hazard ratio; PY=person-year; Rate= (event/person-year) *1000

| **Observation**  **Duration** | **User** | | | **Non-user** | | | **Crude** | **p-value** | **Adjusted** | **p-value** | **Subdistribution** | **p-value** |
| --- | --- | --- | --- | --- | --- | --- | --- | --- | --- | --- | --- | --- |
|  | **Event** | **Total of PY** | **Rate** | **Event** | **Total of PY** | **Rate** | **HR (95% CI)** |  | **HR^a^ (95% CI)** |  | **HR^a^ (95% CI)** |  |
| **One-year confirmed period (n=41163)** | | |  |  |  |  |  |  |  |  |  |  |
| 1 year | 64 | 39807.68 | 1.61 | 88 | 39848.58 | 2.21 | 0.73 (0.53-1.00) | 0.053 | 0.73 (0.50-1.06) | 0.100 | 0.73 (0.51-1.05) | 0.089 |
| 3 years | 166 | 110842.35 | 1.50 | 222 | 111296.77 | 1.99 | 0.75 (0.61-0.92)^**^ | 0.005 | 0.81 (0.64-1.03) | 0.084 | 0.83 (0.66-1.04) | 0.106 |
| 5 years | 242 | 169809.01 | 1.43 | 338 | 171929.91 | 1.97 | 0.72 (0.61-0.85)^***^ | <.001 | 0.76 (0.63-0.93)^**^ | 0.006 | 0.78 (0.65-0.94) ^*^ | 0.010 |
| 7 years | 309 | 216258.33 | 1.43 | 437 | 221099.95 | 1.98 | 0.72 (0.62-0.84)^***^ | <.001 | 0.75 (0.63-0.89)^**^ | 0.001 | 0.77 (0.65-0.91)^**^ | 0.002 |
| 9 years | 360 | 250930.75 | 1.43 | 500 | 258959.43 | 1.93 | 0.74 (0.65-0.85)^***^ | <.001 | 0.75 (0.64-0.88)^***^ | <.001 | 0.77 (0.66-0.91)^**^ | 0.001 |
| End of data | 406 | 288463.76 | 1.41 | 603 | 301795.17 | 2.00 | 0.70 (0.62-0.80)^***^ | <.001 | 0.73 (0.63-0.85)^***^ | <.001 | 0.75 (0.65-0.87)^***^ | <.001 |
| **Three-year confirmed period (n=63457)** | | | |  |  |  |  |  |  |  |  |  |
| 1 year | 99 | 61184.81 | 1.62 | 103 | 61215.07 | 1.68 | 0.96 (0.73-1.27) | 0.781 | 1.06 (0.79-1.43) | 0.683 | 1.07 (0.80-1.45) | 0.647 |
| 3 years | 234 | 168459.92 | 1.39 | 302 | 169839.35 | 1.78 | 0.78 (0.66-0.93)^**^ | 0.005 | 0.81 (0.68-0.98)^*^ | 0.029 | 0.83 (0.69-1.00)^*^ | 0.045 |
| 5 years | 347 | 254106.79 | 1.37 | 447 | 259004.33 | 1.73 | 0.79 (0.69-0.91)^**^ | 0.001 | 0.81 (0.70-0.95)^**^ | 0.008 | 0.83 (0.71-0.97)^*^ | 0.016 |
| 7 years | 445 | 319327.67 | 1.39 | 574 | 328465.06 | 1.75 | 0.80 (0.70-0.90)^***^ | <.001 | 0.81 (0.71-0.92)^**^ | 0.002 | 0.83 (0.72-0.95)^**^ | 0.005 |
| 9 years | 522 | 363840.48 | 1.43 | 667 | 378016.95 | 1.76 | 0.81 (0.73-0.91)^***^ | <.001 | 0.83 (0.73-0.94)^**^ | 0.003 | 0.85 (0.75-0.96)^*^ | 0.011 |
| End of data | 557 | 390773.52 | 1.43 | 720 | 409424.99 | 1.76 | 0.81 (0.73-0.91)^***^ | <.001 | 0.83 (0.73-0.93)^**^ | 0.002 | 0.85 (0.75-0.96)^**^ | 0.007 |

a=Adjusted for age group, gender, insurance premium, arrhythmia, asthma, heart failure, and COPD severity.;

PH=pulmonary hypertension; IRR=incidence rate ratio; aIRR=adjusted incidence rate ratio; PY=person-year; Rate= (Event/person-years)*1000

Supplementary Table 3 Other etiologies of pulmonary hypertension

| **Etiology** | **ICD-9CM code** |
| --- | --- |
| human immunodeficiency virus z | 042 |
| sleep apnea | 327, 780, 786 |
| pulmonary embolism | 4151 |
| connective tissue disease | 446, 701, 710, 711, 719, 714, 720, 725, 728, 729 |
| congenital heart defect | 5723 |

Supplementary Table 4 The definition of baseline characteristics

| **Baseline Characteristics** | **Definition** |
| --- | --- |
| **Age group** | Age when the patient diagnosed COPD |
| 40 ≤ age < 50 |  |
| 50 ≤ age < 60 |  |
| 60 ≤ age < 70 |  |
| 70 ≤ age < 80 |  |
| 80 ≤ age < 90 |  |
| **Gender** | The mode of gender in medical records. The number represented, male is “1” female is “2”. |
| **Insurance premium (TWD$)** |  |
| ≤ 22,800 | The first level of premium is 22,800 TWD dollars. |
| > 22,800 |  |
| **Urbanization level** |  |
| Urban | urbanization level 1 |
| Suburban | urbanization level 2 |
| Rural | urbanization level ≥ 3 |
| **Comorbidity** | **ICD-9 CM/ICD-10 CM** |
| Dyslipidemia | 272/E75, E77-E78 |
| Hypertension | 401-405/I10-I15 |
| Diabetes Mellitus | 250/E10-E14 |
| Obesity | 278.0/E661 E663 E668 E669 E6601 E662 |
| Chronic kidney disease | 585/ N184 N185 N186 N189 |
| Chronic liver disease | 070.22, 070.23, 070.32, 070.33, 070.54, 070.59, 070.6, 070.9, 456.0, 456.1, 456.2, 570, 571, 572.2, 572.3, 572.4, 572.8, 573.3, 573.4, 573.8, 573.9, V42.7/K70, K73-74 |
| Arrhythmia | 427-426/I44-I49, R001 |
| Interstitial pulmonary diseases | 4293/J84 |
| Asthma | 493/J45 |
| Malignant | 140-239/C00-D49 |
| **ASCVD** |  |
| Coronary artery disease | 410-414, 429.2/I20-I25 |
| Peripheral vascular disease | 440-448/I70-I70, M30-M31 |
| Ischemic stroke/ TIA | 433-437/ G45-G46, I63-I68 |
| Hemorrhagic stroke | 430-432/I60-I62 |
| Heart failure | 398.91, 402.01, 402.11, 402.91, 404.01, 404.03, 404.11, 404.13, 404.91, 404.93, 425.4, 425.5, 425.7, 425.8, 425.9, 428/I50 |
| Left ventricular hypertrophy | 4293/ I517 |
| **Co-medication** | **Drugs** |
| **Nonstatins lipid-lowering drugs** | |
| fibrates | clofibrate, bezafibrate, aluminium clofibrate, gemfibrozil, fenofibrate, simfibrate, etofibrate |
| Others (Contain nicotinic acid and derivatives, bile acid and PCSK9 inhibitors) | niceritrol, nicotinic acid, nicofuranose, acipimox, nicomol, colestyramine, colestipol, colextran, probucol, ezetimibe, evolocumab |
| **Other medications** | |
| digoxin | digitoxin, digoxin, metildigoxin |
| oral anticoagulant agents | warfarin, dabigatran etexilate, rivaroxaban, apixaban, edoxaban |
| oral antiplatelet agents | clopidogrel, ticlopidine, acetylsalicylic acid, dipyridamole, prasugrel, cilostazol, ticagrelor |
| Diuretics | There are too many types, so those did not list here. |
| ACEI/ACB | There are too many types, so those did not list here. |
| beta blocker | There are too many types, so those did not list here. |
| metformin | metformin |
| **Specific drug therapy of PH** | |
| calcium channel blockers | diltiazem, amlodipine, felodipine, isradipine, nicardipine, nifedipine, nimodipine, nisoldipine, nitrendipine, lacidipine, barnidipine, lercanidipine, benidipine |
| endothelin receptor antagonists | ambrisentan, bosentan, macitentan |
| phosphodiesterase type 5 inhibitors and guanylate cyclase stimulators | alprostadil, sildenafil, riociguat |
| prostacyclin analogues and prostacyclin receptor agonists | beraprost, epoprostenol, iloprost, treprostinil, |
| **Medication for COPD** | |
| LABA | salmeterol, formoterol, indacaterol, olodaterol |
| LABA/ICS | almeterol and fluticasone, formoterol and budesonide, vilanterol and fluticasone furoate |
| LAMA | tiotropium bromide, glycopyrronium bromide, umeclidinium bromide |
| LABA/LAMA | vilanterol and umeclidinium bromide, indacaterol and glycopyrronium bromide, olodaterol and tiotropium bromide |
| SABA | salbutamol, terbutaline, fenoterol, procaterol |
| SAMA | ipratropium bromide |
| SABA/SAMA | fenoterol and ipratropium bromide, salbutamol and ipratropium bromide |
| systemic beta-2-adrenoreceptor agonists | salbutamol, terbutaline, fenoterol, hexoprenaline, procaterol, tretoquinol, bambuterol, clenbuterol |
| ICS | beclometasone, budesonide, fluticasone, ciclesonide |
| methyl-xanthines | diprophylline, choline theophyllinate, proxyphylline, |

COPD=chronic obstructive pulmonary disease; PH=pulmonary hypertension; TWD= Taiwan dollars; ASCVD=atherosclerotic cardiovascular disease; TIA=Transient ischemic attack; CCB=calcium channel blocker; ACEI=angiotensin converting enzyme inhibitor; ARB=angiotensin receptor blocker; LABA= Long-acting β_2_-aginist; LAMA=Long-acting muscarinic antagonists; SABA= Short-acting β_2_-aginist; SAMA= Short-acting muscarinic antagonists; ICS= Inhaled corticosteroid; ICD-9CM=International Classification of Diseases, Ninth Revision, Clinical Modification
